# Mapping glucose-mediated gut-to-brain signalling pathways in humans^☆^

**DOI:** 10.1016/j.neuroimage.2014.03.059

**Published:** 2014-08-01

**Authors:** Tanya J. Little, Shane McKie, Richard B. Jones, Massimo D'Amato, Craig Smith, Orsolya Kiss, David G. Thompson, John T. McLaughlin

**Affiliations:** a University of Manchester, Manchester Academic Health Sciences Centre (MAHSC), Gastrointestinal Centre, Institute of Inflammation and Repair, Faculty of Medical and Human Sciences, University of Manchester, Clinical Sciences Building, Salford Royal Hospital, Stott Lane, Salford, UK, M6 8HD, UK; b University of Manchester, Manchester Academic Health Sciences Centre (MAHSC), Neuroscience and Psychiatry Unit, Institute of Brain, Behaviour and Mental Health, The University of Manchester, G907 Stopford Building, Oxford Road, Manchester M13 9PT, UK; c Rottapharm SpA, Via Valosa di Sopra, 20900 Monza, Italy; d Faculty of Life Sciences, The University of Manchester, Manchester M13 9PT, UK

**Keywords:** (CCK), cholecystokinin, (physMRI), physiological magnetic resonance imaging, (GE), gastric emptying, (GI), gastrointestinal, (GLP-1), glucagon-like peptide-1, (CNS), central nervous system, Glucose, Cholecystokinin (CCK), Dexloxiglumide, physMRI, nutrient

## Abstract

**Objectives:**

Previous fMRI studies have demonstrated that glucose decreases the hypothalamic BOLD response in humans. However, the mechanisms underlying the CNS response to glucose have not been defined. We recently demonstrated that the slowing of gastric emptying by glucose is dependent on activation of the gut peptide cholecystokinin (CCK_1_) receptor. Using physiological functional magnetic resonance imaging this study aimed to determine the whole brain response to glucose, and whether CCK plays a central role.

**Experimental design:**

Changes in blood oxygenation level-dependent (BOLD) signal were monitored using fMRI in 12 healthy subjects following intragastric infusion (250 ml) of: 1 M glucose + predosing with dexloxiglumide (CCK_1_ receptor antagonist), 1 M glucose + placebo, or 0.9% saline (control) + placebo, in a single-blind, randomised fashion. Gallbladder volume, blood glucose, insulin, and GLP-1 and CCK concentrations were determined. Hunger, fullness and nausea scores were also recorded.

**Principal observations:**

Intragastric glucose elevated plasma glucose, insulin, and GLP-1, and reduced gall bladder volume (an *in vivo* assay for CCK secretion). Glucose decreased BOLD signal, relative to saline, in the brainstem and hypothalamus as well as the cerebellum, right occipital cortex, putamen and thalamus. The timing of the BOLD signal decrease was negatively correlated with the rise in blood glucose and insulin levels. The glucose + dex arm highlighted a CCK_1_-receptor dependent increase in BOLD signal only in the motor cortex.

**Conclusions:**

Glucose induces site-specific differences in BOLD response in the human brain; the brainstem and hypothalamus show a CCK_1_ receptor-independent reduction which is likely to be mediated by a circulatory effect of glucose and insulin, whereas the motor cortex shows an early dexloxiglumide-reversible increase in signal, suggesting a CCK_1_ receptor-dependent neural pathway.

## Highlights

• We have identified two distinct CNS responses to glucose in man.
• A CCK1 receptor (CCK1R)-dependent BOLD signal increase in the motor cortex.
• A CCK1R-independent BOLD signal decrease in the brainstem and hypothalamus.
• The BOLD signal decrease was mediated by changes in blood glucose and insulin,

## Introduction

Obesity is now one of the major public health problems facing the world. In 2005, 400 million adults were obese, and this is projected to increase to 700 million by 2015 (www.who.int). This “epidemic” has prompted major scientific efforts to determine the mechanisms underlying the regulation of food intake in order to facilitate the development of effective therapies for obesity. A particular focus has been applied to nutrient-induced gut-to-brain signalling mechanisms. In human research, the development of blood-oxygenation-level-dependent (BOLD) functional magnetic resonance imaging (fMRI) has resulted in the identification of a number of homeostatic and non-homeostatic brain regions associated with the regulation of appetite (Gibson et al., 2010). However, to date, the focus of these human studies has largely been the evaluation of higher centre cognitive responses to food cues (such as images of palatable food), rather than the physiological responses to meal ingestion. The more recent application of the physiological MRI (physMRI) technique (Jones et al., 2012, Lassman et al., 2010) has allowed the exploration of BOLD signal responses to nutrients over time across the whole brain, in particular, in key areas involved in the regulation of food intake such as the brainstem and hypothalamus.

It is well established that the presence of glucose in the small intestinal lumen is associated with decreased hunger, increased fullness, and decreased energy intake at a subsequent meal (Cook et al., 1997, Lavin et al., 1996, Pilichiewicz et al., 2007), but the brain centres and transduction mechanisms underlying these effects are poorly defined in humans. Animal studies have identified the hypothalamus as a critical region for the regulation of energy balance (Brown and Melzack, 1969, Grossman, 1975), and in man, a number of fMRI studies have reported that oral glucose decreases hypothalamic BOLD signal, relative to water (Liu et al., 2000, Smeets et al., 2005a, Smeets et al., 2005b, Smeets et al., 2007, Vidarsdottir et al., 2007). However, these studies did not investigate the responses of other sub-cortical brain regions to glucose, and the movement of the head during consumption of oral meals induced imaging artefacts that precluded evaluation of the early responses. In particular, detailed imaging of the human brainstem has not been reported and is critical as it is the first relay point responsible for integrating neural signals transmitted from the upper gut via vagal afferent neurons.

The mechanism by which the presence of glucose in the gut is signalled to the human central nervous system is likely to involve an interaction between direct responses to elevated circulating glucose concentrations (Levin et al., 2004), and indirect responses via glucose sensitive neural (e.g. vagal), or endocrine signalling pathways (e.g. insulin (Liu et al., 2000, Smeets et al., 2005b) and/or gut derived peptides, such as cholecystokinin (CCK) (Lassman et al., 2010, Little et al., 2010) and glucagon-like peptide-1 (GLP-1) (Pannacciulli et al., 2007)). Recent studies have indicated that signals arising from the gastrointestinal tract are likely to play an important role in mediating the hypothalamic response to glucose. For example, the effect of glucose on hypothalamic BOLD signal has been reported to be greater when administered orally compared to intravenously (Smeets et al., 2007). The hypothalamic response has also been related to fasting insulin concentrations (Liu et al., 2000, Matsuda et al., 1999), and additionally a relationship has been found between postprandial plasma GLP-1 concentrations and increases in BOLD signal in the left dorsolateral prefrontal cortex and the hypothalamus, following ingestion of a liquid meal (Pannacciulli et al., 2007). However, in these previous studies, whole brain responses, and time courses of the response could not be evaluated.

Using physMRI to investigate whole brain responses, we have previously shown that intragastrically administered lipid (the fatty acid, lauric acid (C12)) increases BOLD signal in key human brain areas, including the brainstem and hypothalamus (Lassman et al., 2010). These activations were abolished by the administration of the CCK_1_ receptor antagonist, dexloxiglumide (Lassman et al., 2010), and modulated by ghrelin (Jones et al., 2012).

We, therefore, decided to apply the same imaging technique to identify and define the brain regions responsive to glucose. Like lipid, glucose ingestion is known to slow gastric emptying (Hunt and Knox, 1968), and to induce gallbladder contraction (de Boer et al., 1993). In addition, dexloxiglumide attenuates the inhibitory effects of glucose on gastric emptying (Fried et al., 1991, Little et al., 2010), suggesting that CCK is also involved in this, and possibly mediating the gut-to-brain signalling response to glucose. Therefore, the CNS response to different macronutrients may be predicted to have significant overlaps.

This study therefore had two aims: first, to determine whether intragastric glucose would induce changes in BOLD signal in the same nutrient-sensitive regions, robustly observed in response to lipid, namely the brainstem and hypothalamus, and second, to determine whether any effects of glucose observed in the CNS were also CCK_1_ receptor-dependent.

## Methods

### Subjects

12 healthy volunteers (7 males, mean (SEM) age: 38 (3.4) (range: 23–60) years) with a non-obese body mass index (BMI: 24 (1) (range: 19.7–28.9) kg/m^2^) were recruited from the staff and local population of Salford Royal NHS Foundation Trust. Exclusion criteria comprised: presence of any gastrointestinal, endocrine or metabolic disease; consumption of medications that may affect gastrointestinal function, sensation or appetite; metallic implants or objects (e.g. hearing aid, pacemaker), and consumption of greater than 20 g of alcohol or 10 cigarettes/day. All were instructed to abstain from alcohol and vigorous physical activity for 24 h prior to the study. In both the gastric emptying and the fMRI study arms, participants were blinded both to the nature of the intragastric infusions, and to the drug, and studies were performed in a randomised order. Randomisation was performed using a random number generator. Participants provided informed written consent. The study was approved by the Salford and Trafford Research Ethics Committee, and was carried out in accordance with the Code of Ethics of the World Medical Association (Declaration of Helsinki) and the standards established by the author's ethics committee and granting agency. All participants provided informed, written consent after the nature of the experimental procedures provided in writing.

### Materials

d-Glucose was purchased from Sigma-Aldrich (Gillingham, England). The CCK_1_ receptor antagonist, dexloxiglumide (300 mg tablets), and matched placebo tablets were supplied at no cost by Rottapharm (Monza, Italy) as a scientific collaboration.

### Gastric emptying protocol

As the medulla houses the sensory and motor nuclei responsible for relaying vago-vagal enteric circuits, as well as signalling to higher centres, we used the measurement of changes in gastric emptying induced by glucose as a convenient proxy measure to identify a dose of glucose that would have effects on brainstem BOLD response (Little et al., 2010). Participants arrived at the laboratory at 09:00 h after an overnight fast from 22:00 h and orally ingested either 600 mg of dexloxiglumide (1 occasion), or matched placebo (2 occasions), tablets. Previous studies in our laboratory have demonstrated that this dose effectively blocks the effects of lipid (lauric acid (C12)) (Lal et al., 2004) and glucose (Little et al., 2010) on gastric emptying, and the effect of lipid on BOLD signal in the brainstem and hypothalamus (Lassman et al., 2010). One hour later, a nasogastric feeding tube was positioned, with its distal tip in the stomach. Gastric delivery was employed to eliminate sensory or hedonic effects of oral sweet tasting, and for the MRI studies, movement artefacts during drinking and swallowing. A basal, end-expiratory, breath sample was collected; following which participants received a bolus intragastric infusion of 250 ml glucose (45 g, 180 kcal, 1000 mOsmol) or 250 ml of water (volumetric control). Each meal was labelled with 100 mg ^13^C-sodium acetate, used as a marker for gastric emptying (CK Gas Products Ltd., UK). For the assessment of gastric emptying rate, end-expiratory breath samples were collected at 5 minute intervals over a period of 45 min (Little et al., 2009, Little et al., 2010). On each occasion, the subject exhaled through a mouth-piece to collect an end-expiratory breath sample into a 100 ml foil bag, which was then sealed with a plastic stopper, and stored for later analysis.

Breath samples were analysed by non-dispersive infrared spectroscopy (IRIS, Wagner Analysen Technik, Bremen, Germany). This enabled evaluation of the isotopic composition of carbon in the expired carbon dioxide (CO_2_) by determination of the ratio of ^13^C:^12^C in the sample (Lee et al., 2000, Sanaka et al., 2006). ^13^C-acetate is rapidly absorbed and metabolized after it is emptied from the stomach, hence the rate of appearance of ^13^CO_2_ in the systemic circulation closely corresponds to the rate of GE (Sanaka et al., 2006).

### physMRI protocol

12 participants were studied on three separate occasions at 08:30 h following an overnight fast from 22:00 h, to evaluate the spatiotemporal pattern of brain activation in response to intragastric infusion of: (i) glucose (250 ml, 1000 mOsmol (45 g glucose, 180 kcal)) following two placebo tablets, (ii) glucose (250 ml, 1000 mOsmol) following the CCK_1_ receptor antagonist, dexloxiglumide (600 mg (two 300 mg tablets)) (glucose + dex), and (iii) 0.9% saline (control) following two placebo tablets. The dexloxiglumide (condition ii), or matched placebo, tablets (conditions i and iii), were orally ingested with 100 ml of water 1 h prior to the commencement of the intragastric infusion. All conditions were matched exactly to our previous studies (Jones et al., 2012, Lassman et al., 2010) to enable direct comparison between the glucose- and lipid-induced nutrient matrices. In the study design we purposefully omitted a saline + dex control arm since we had previously demonstrated, under the same study conditions (i.e. after an overnight fast and with identical imaging parameters), that there was no effect of dexloxiglumide in the absence of nutrient (i.e. during intragastric saline infusion) on BOLD signal across the whole brain (Lassman et al., 2010). Furthermore, dexloxiglumide has no effect on the gastric emptying of water/saline (Little et al., 2010). Due to the large number of statistical comparisons to be performed, which do not require the placebo/placebo condition, and mindful of burden on experimental subjects, we considered that repeating this was unjustified.

Thirty minutes prior to scanning, participants were intubated with a nasogastric feeding tube and an intravenous cannula was positioned in an antecubital vein to enable repeated blood sampling. Participants were then positioned in the scanner (3.0 T Philips Achieva MR System, Magnetic Resonance Imaging Facility (MRIF), Salford Royal NHS Foundation Trust) and images of the gallbladder acquired. Immediately following this, participants were repositioned in the scanner for the 30 minute fMRI scan. After a baseline period of 4 min, the intragastric infusion (glucose or saline) was administered as a bolus, using a syringe via the nasogastric tube, over a period of 1 min. Subjective ratings of fullness, hunger and nausea were recorded every 5 min throughout the scan. Venous blood samples were taken to assess blood glucose, and plasma insulin, CCK and GLP-1 concentrations at the start of the scan protocol (t = 0 min), and then at t = 5, 10, 15, 20, and 25 min following the intragastric infusion. After the fMRI scan, a second gallbladder image was acquired. On the first visit, participants were positioned in the scanner an additional 10 min before the commencement of the scanning protocol to enable a high-resolution structural scan to be performed to more accurately normalise each individual's brain anatomy.

### Gallbladder MR acquisition and volume measurement

Gallbladder volume, which is a classical *in vivo* bioassay for CCK activity, was determined to evaluate the functional significance of the CCK response to the glucose test meals and as a positive control for CCK_1_ receptor blockade by dexloxiglumide (Lilja et al., 1982). Complete blockade of gallbladder contraction demonstrates effective CCK_1_ receptor antagonism. Volumes were determined from single-shot, T2 weighted axial, coronal and sagittal 3 T anatomical MR images of the upper abdomen obtained at the beginning and end of each study using a SENSE torso coil triggered by respiration. Gallbladder volume was estimated using the ellipsoid method, whereby volume = length × depth × width × 0.523 (where π/6 = 0.523, depth = maximum antero-posterior diameter and width = maximum lateral diameter) (Dodds et al., 1985, Lassman et al., 2010).

### Sensation scores

Subjects were asked to indicate their sensations of hunger, fullness and nausea on a 10 point scale, every 5 min during each scan. The subject rated each sensation by moving a pointer along the scale, via a response box held in their right hand (Lassman et al., 2010).

### Blood glucose, plasma insulin, CCK and GLP-1 measurements

Venous blood samples (10 ml) were collected into ice-chilled lithium heparin-treated tubes containing 400 kIU aprotinin per ml blood (Trasylol; Bayer Ltd., Berkshire, UK). Plasma was separated by centrifugation at 3000 rpm for 10 min at 4 °C within 1 h of collection, and stored at − 70 °C until assayed.

Venous blood glucose concentrations (mmol/l) were determined at the time of collection by the glucose oxidase method using a portable glucose meter (Medisense Optium Xceed, Abbott, Alameda CA USA). The accuracy of this method has been confirmed using the hexokinase technique (Horowitz et al., 1991).

Plasma insulin concentrations were determined using the ALPCO Insulin ELISA kit (ALPCO, Product number: 80-INSHU-E01.1, Salem, NH, USA). The sensitivity of the assay is 0.399 uIU/ml. The intra-assay coefficients of variability (CVs) were 5.1–10.3%, and the inter-assay CVs were 6.7–16.6%.

Plasma GLP-1 concentrations were determined using the ALPCO GLP-1 (7–36) and GLP-1 (9–36) ELISA kit (ALPCO, Product number: 43-GPTHU-E01, Salem, NH, USA). The assay has 0.01% cross-reactivity with GLP-1 and glucagon. The sensitivity of this assay is 0.06 pmol/l. The intra-assay CVs were 3.7–4.7%, and the inter-assay CVs were 6.2–9.5%.

Plasma CCK concentrations were determined using the ALPCO EURIA-CCK radioimmunoassay kit (ALPCO, Product number: 13-RB302). The assay has 0.5% cross-reactivity with gastrin-17 sulphated, and < 0.01% with gastrin-17 non-sulphated. The sensitivity of the assay is 0.3 pmol/l. The intra-assay CVs were 2–5.5%, and the inter-assay CVs were 4.1–13.7%.

### MRI acquisition

fMRI brain volumes were acquired at 5 second intervals for 30 min using a multi-slice, single-shot EPI sequence to achieve whole brain coverage: 40 axial slices, TR/TE = 5000/35 ms, voxel size = 1.8 × 1.8 × 3.5 mm, flip angle = 79°, SENSE acceleration factor = 1.8, matrix = 128 × 128, FOV = 240 × 240 mm. Daily quality assurance was carried out to ensure a consistent high signal-to-ghost ratio using an automated quality control procedure. A SENSE 8 channel head coil was used for radio frequency transmission and reception.

### MRI image analysis

The fMRI data were analysed using Statistical Parametric Mapping (SPM8, www.fil.ion.ucl.ac.uk/spm). Images were realigned, spatially normalised and smoothed to facilitate inter-subject averaging. The limits on head movement for inclusion were: 2 mm for x- and y-translation and 4 mm for z-translation, both per volume and across the whole scan, and 3° for yaw and 1.5° for roll and pitch, again per volume and across the whole scan.

First-level analysis was performed, using the p-block ph/physMRI analysis technique (Jones et al., 2012, Lassman et al., 2010, McKie et al., 2005, McKie et al., 2011), on each subject for each study condition in the following ways: the pre- and post-infusion scans were split into two minute time-bins. The post-infusion scans were divided into 13 consecutive 2 minute time bins (T1–13) and the 24 scans from the 2 min immediately prior to the glucose/saline infusion formed the baseline time bin (T0). In each subject and condition, the signal averages for the 13 post-infusion time bins (T1–T13) were separately compared to the baseline average (T0) using regression within the general linear model framework. This resulted in 13 first level images corresponding to the BOLD signal change from baseline in each successive post-infusion time bin for each subject and condition. The realignment parameters for each session were included as regressors of no interest to correct for motion. Scanner drift was modelled using the scaling option for global normalisation in SPM. Contrast maps across time for glucose vs. saline and glucose vs. glucose + dex were calculated for each subject.

To determine whether statistically significant increments in the BOLD signal change from baseline across subjects occurred over time, two repeated-measures ANOVAs were used, one for the glucose vs. saline comparison and the other for the glucose vs. glucose + dex comparison, at a cluster-level statistical inference of p(False Discovery Rate; FDRc) < 0.05 at a height threshold of p = 0.005.

Correlations were performed to determine relationships between changes in BOLD signal with blood glucose, plasma GLP-1 and insulin concentrations, and the subjective ratings of nausea, hunger and fullness. These were performed using the average values per subject (area under the curve (AUC) and across time) for each significant cluster extracted from the glucose vs. saline and glucose + dex vs. saline comparisons. For the AUC correlations, all time bin BOLD signal change contrasts were averaged per cluster and compared to the baseline corrected AUC blood concentrations for each individual. For the correlations with time, each cluster's average BOLD signal at five time bins (T + 5, T + 10, T + 15, T + 20 and T + 25 min) was calculated and correlated to the each subject's blood concentration measures at the same time points. This resulted in a correlation coefficient, r, for each subject. Each subject's r-values were then entered into a one-sample *t*-test. All correlations are reported at a False Discovery Rate level (pFDR < 0.05) correcting for the number of cluster significant regions being tested.

### Analysis of gastric emptying, blood glucose and GI hormone concentrations and appetite perceptions

Gastric emptying, blood glucose, plasma CCK, GLP-1 and insulin concentrations are displayed as raw data. VAS scores were analysed as change from baseline values. Areas under the curve were calculated for gastric emptying and blood glucose, CCK, GLP-1 and insulin concentrations using the trapezoidal rule. These data were analysed using repeated measures ANOVAs with treatment, and time (or for the AUC data, treatment) as factors. If ANOVAs revealed statistical significance, post-hoc paired comparisons, corrected for multiple comparisons using Bonferroni's correction, were performed. Statistical analysis was performed using SPSS Version 17.0. Data are presented as means (SEM).

## Results

The study was well tolerated by all participants. Due to difficulty obtaining blood samples from 2 of the participants while in the scanner, complete data sets for glucose, insulin, CCK and GLP-1 are available from only 10 participants. Three participants self-reported smoking ≤ three cigarettes/day. There were no apparent differences in gastric emptying, BOLD response or gut peptide secretion between the smokers and non-smokers, but this could not be formally evaluated due to the small numbers.

### Gastric emptying

**Fig. 1:**
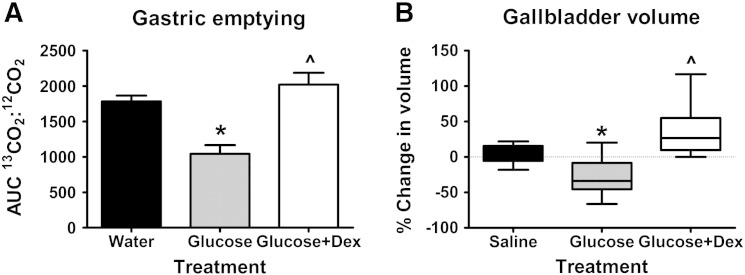
(A) Gastric emptying (n = 3/12) and (B) change in gallbladder volume (n = 12) following intragastric glucose (1000 mOsmol), glucose (1000 mOsmol) + dexloxiglumide (600 mg) or saline/water control. Data are mean (SEM). *glucose vs. water/saline, ^glucose + dex vs. glucose, p < 0.05.

As in our previous study (Little et al., 2010), we demonstrated an effect of treatment on gastric emptying (p = 0.003). Intragastric glucose slowed gastric emptying when compared with water (p = 0.017), and dexloxiglumide abolished this glucose-induced inhibition (p = 0.031), with no difference between the emptying of water and glucose + dex. The slower gastric emptying observed after glucose consumption is therefore CCK_1_-receptor dependent (Fig. 1A).

### Gallbladder volumes

There was an effect of intragastric glucose on gallbladder volume (Fig. 1B). Glucose decreased the volume of the gallbladder (p = 0.014) when compared with saline. As with gastric emptying, the effect of glucose was attenuated by dexloxiglumide pre-treatment (p = 0.008). This provides evidence of fully effective CCK_1_ receptor blockade by dexloxiglumide.

### Areas of glucose-induced BOLD signal change

**Fig. 2:**
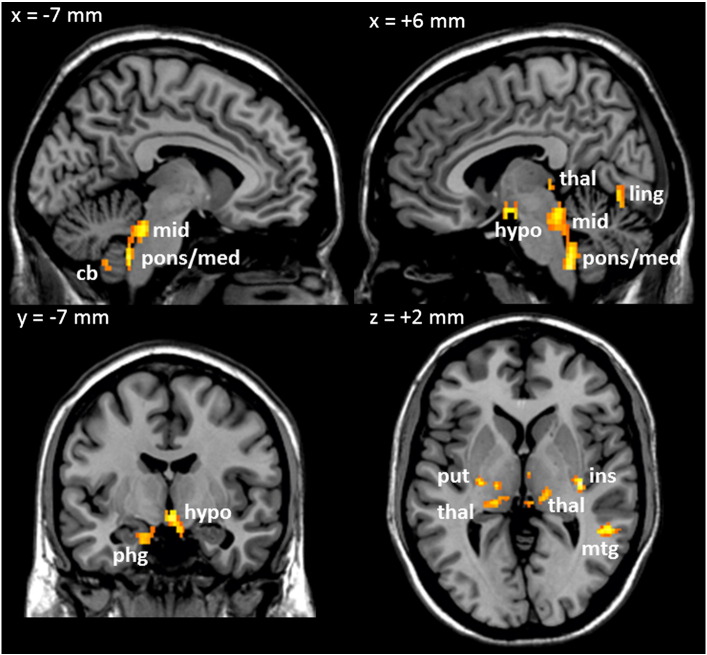
Brain images showing areas exhibiting significant effect of time (pFDRc < 0.05 at p < 0.005) for glucose versus saline contrast. cb: cerebellum, pons/med: pons/medulla, mid: midbrain, hypo: hypothalamus, thal: thalamus, ling: lingual gyrus, phg: parahippocampal gyrus, put: putamen, ins: insula, mtg: middle temporal gyrus.

Results of the one-way ANOVAs investigating the differences in BOLD signal responses over time following intragastric glucose (relative to saline) are presented in Table 1 and Fig. 2, Fig. 4. A significant effect of time was observed in sections of the brainstem (Figs. 4A, B, C) and hypothalamus (Fig. 4D) (regions previously reported to show a positive BOLD response between lipid and saline (Jones et al., 2012, Lassman et al., 2010)), as well as the cerebellum, right fusiform and lingual gyri, insula/putamen, left parahippocampal gyrus, right middle temporal and thalamic regions. Investigation of the average time courses for these significant clusters highlighted that the effect of time was generally driven by an initial increase in BOLD signal followed by sharp decrease at 5 min (Fig. 4).

**Table 1:** Significant central nervous system clusters exhibiting effect of time between glucose and saline infusions: p(FDRc < 0.05) at p = 0.005. TD — Talairach Daemon; pFDRc — cluster size False Discovery Rate p-value; CoM — centre of mass; x,y,z {mm} — Montreal Neurological Institute (MNI) coordinates from anterior commissure origin; wrt — with respect to.

Cluster-level statistics		(Glucose vs saline) ∗ time	CoM			Glucose vs saline correlation wrt time		Glucose + dex vs saline correlation wrt time	
						Blood glucose	Insulin	Blood glucose	Insulin
Size at p < 0.005	pFDRc	TD lobes & hypothalamus	x,y,z {mm}			pFDR		pFDR	
61	0.045	Brainstem–medulla L + R	1	− 43	− 47	0.034	0.055	ns	ns
77	0.023	Brainstem–pons L + R	3	− 39	− 36	0.030	0.055	ns	ns
121	0.004	Brainstem–midbrain L + R	2	− 34	− 19	0.013	0.026	ns	ns
104	0.007	Hypothalamus L + R	2	− 3	− 12	0.007	0.004	ns	ns
111	0.006	Cerebellum–anterior L + R	− 2	− 42	− 33	0.012	0.019	ns	ns
357	< 0.001	Cerebellum L	− 23	− 56	− 41	0.012	0.019	0.062	0.042
177	< 0.001	Cerebellum R	17	− 52	− 22	0.026	0.029	0.070	0.037
95	0.011	Fusiform_R	30	− 73	− 9	0.012	0.017	0.026	0.005
76	0.024	Lingual_R	10	− 71	− 7	ns	ns	0.050	0.009
42	0.099	Insula_L/putamen_L	− 27	− 15	7	ns	ns	ns	ns
68	0.033	Insula_R/putamen_R	35	− 17	2	ns	ns	ns	ns
71	0.030	Parahippocampal_L	− 17	− 3	− 22	ns	ns	ns	0.049
59	0.049	Temporal_Mid_R	53	− 45	5	ns	ns	ns	ns
60	0.047	Thalamus_L	− 17	− 28	3	0.058	ns	ns	ns
47	0.080	Thalamus_R	11	− 26	1	0.029	ns	ns	ns

### Effect of dexloxiglumide on areas of BOLD signal change

**Fig. 3:**
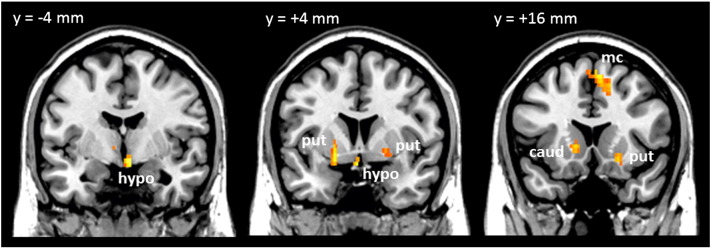
Brain images showing areas exhibiting significant effect of time (pFDRc < 0.05 at p < 0.005) for glucose versus glucose + dex contrast. hypo: hypothalamus, put: putamen, caud: caudate, mc: motor cortex (SMA).

**Fig. 4:**
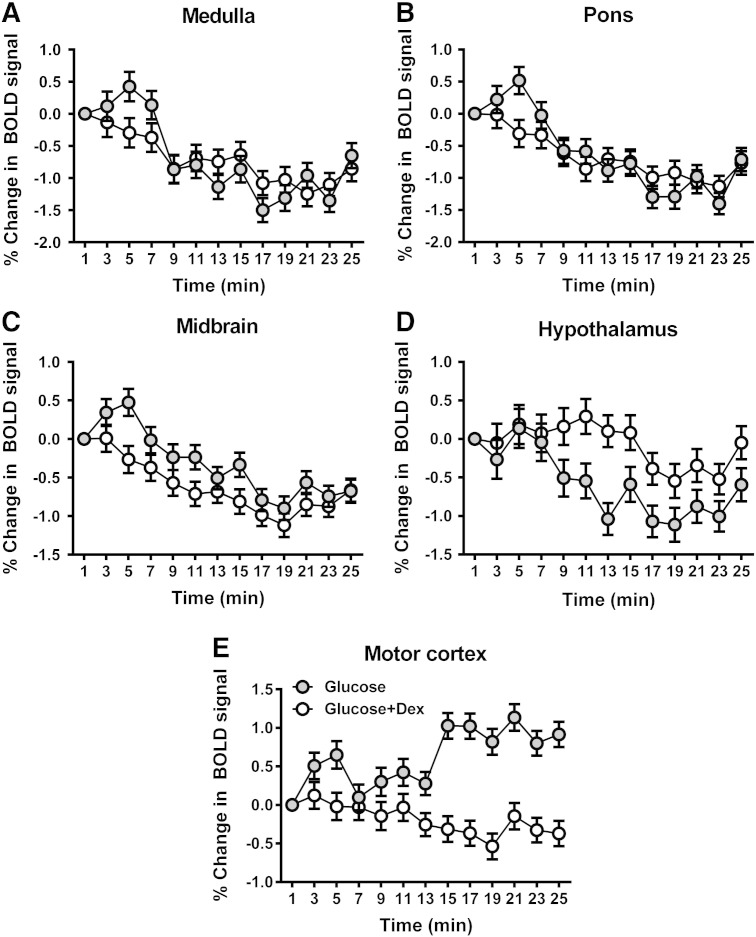
Changes in blood-oxygenation-level-dependent (BOLD) signal over time in the medulla (A), pons (B), midbrain (C), and hypothalamus (D), and motor cortex (E), following bolus intragastric glucose (1000 mOsmol) and glucose + dex, relative to saline.

Dexloxiglumide pre-treatment significantly modulated the BOLD signal response over time in the regions summarised in Table 2 and Fig. 3. Investigation of the average time courses for the significant clusters highlighted partial blockade of the effect of glucose to decrease BOLD signal in the hypothalamus (Fig. 4D). The supplementary motor area is of particular interest as this was the only region to show a positive BOLD response to glucose ingestion (glucose vs. saline) over time, an effect that was abolished by dexloxiglumide (Fig. 4E). Investigation of the average time course in the other brain regions in Table 2 showed that effect of time was driven in two separate ways: 1) for the cerebellum, left caudate, left and right cuneus, and left lingual gyrus, the effect of time was driven by the BOLD response for glucose + dex vs. saline being further reduced relative to glucose vs. saline, and 2) for the other regions the effect of time was driven by the initial glucose-induced increase in BOLD signal and subsequent sharp decrease at 5 min being abolished by dexloxiglumide pretreatment.

**Table 2:** Significant central nervous system clusters exhibiting effect of time between glucose infusion and glucose infusion following pretreatment with dexloxiglumide p(FDRc < 0.05) at p = 0.005. TD — Talairach Daemon; qFDRc — cluster size False Discovery Rate q-value; CoM — centre of mass; x,y,z {mm} — Montreal Neurological Institute (MNI) coordinates from anterior commissure origin; wrt — with respect to.

Cluster-level statistics		(Glucose vs glucose + dex)*time	CoM			Glucose vs saline correlation wrt time		Gluc + dex vs saline correlation wrt time		
						Blood glucose	Insulin	Blood glucose	Insulin	glp-1
Size at p < 0.005	pFDRc	TD lobes & hypothalamus	x,y,z {mm}			pFDR		pFDR		
71	0.031	Hypothalamus L + R	4	− 1	− 13	0.005	0.004	ns	ns	ns
1027	0.000	Cerebellum L + R	− 2	− 53	− 23	0.031	0.048	ns	0.026	0.045
93	0.014	Caudate_L	− 14	21	0	ns	ns	ns	ns	ns
119	0.005	Cingulum_Mid_L	− 10	− 27	37	ns	ns	ns	ns	ns
62	0.043	Cuneus_L	− 14	− 76	31	ns	ns	ns	0.024	0.022
150	0.002	Cuneus_R	13	− 78	27	ns	ns	ns	0.035	0.016
119	0.005	Insula_L/Putamen_L	− 39	− 18	8	ns	ns	ns	ns	ns
89	0.016	Insula_R/Putamen_R	22	11	− 7	0.003	0.011	ns	ns	ns
184	< 0.001	Lingual_L	− 15	− 68	− 7	0.030	0.038	ns	0.035	0.030
80	0.022	Occipital_Inf_R	36	− 76	− 8	ns	ns	ns	ns	ns
104	0.009	Parahippocampal_L	− 16	− 37	− 1	ns	ns	ns	ns	ns
72	0.030	Parietal_Sup_L	− 18	− 59	45	ns	ns	ns	ns	ns
65	0.039	Parietal_Sup_R	16	− 65	52	ns	ns	ns	ns	ns
71	0.031	Precuneus_L	− 13	− 52	17	0.029	0.045	ns	ns	ns
127	0.004	Precuneus_R	14	− 68	46	ns	ns	ns	ns	ns
63	0.042	Supp_Motor_Area_L + R	7	15	53	ns	ns	ns	ns	ns
130	0.004	Temporal_Mid_R	52	− 47	4	ns	ns	ns	ns	ns
63	0.042	Thalamus_L	− 17	− 27	5	ns	ns	ns	ns	ns

### Time course of changes in BOLD signal

There were significant correlations between the time course of BOLD signal changes with the time course of changes in circulating blood glucose and insulin concentrations in all of the brainstem, hypothalamic, cerebellar, fusiform and thalamic regions responding to glucose (Table 1). This temporal correlation was removed by pretreatment with dexloxiglumide in all of these regions, apart from the lateral cerebellum and the right occipital cortex (fusiform and lingual gyrus). A temporal correlation between the glucose + dex vs. saline BOLD signal with insulin was also observed in the left parahippocampal region. There were no significant correlations between BOLD signal changes with the AUC of blood glucose, GI hormones or subjective appetite measures. There were no significant temporal correlations between BOLD signal changes with GLP-1, or any of the subjective appetite measures.

With respect to clusters that showed a significant modulation of the glucose-induced BOLD signal by dexloxiglumide, there were correlations between the time course of the BOLD signal change with the time course of blood glucose and insulin concentrations in the hypothalamus, cerebellum, right putamen, left lingual gyrus and left precuneus (Table 2). There were no temporal correlations between the time course for blood glucose and the time course for the glucose + dex vs. saline BOLD signal in any region, however, there were temporal correlations observed with insulin and GLP-1 in the cerebellum, bilateral cuneus and left lingual gyrus i.e. the clusters that showed an the effect of time driven by the BOLD response for glucose + dex vs. saline being further reduced in comparison to glucose vs. saline. Interestingly, in the supplementary motor area, where BOLD signal increased in response to glucose (and where the effect was reversed by dexloxiglumide), there was no temporal correlation with circulating blood glucose, insulin or GLP-1 concentrations (Table 2). There were no significant correlations between BOLD signal changes and the AUC of blood glucose, hormones or subjective appetite measures. There were no significant temporal correlations with BOLD for any of the subjective appetite measures.

### Blood glucose, plasma insulin, CCK and GLP-1 concentrations

#### Glucose

**Fig. 5:**
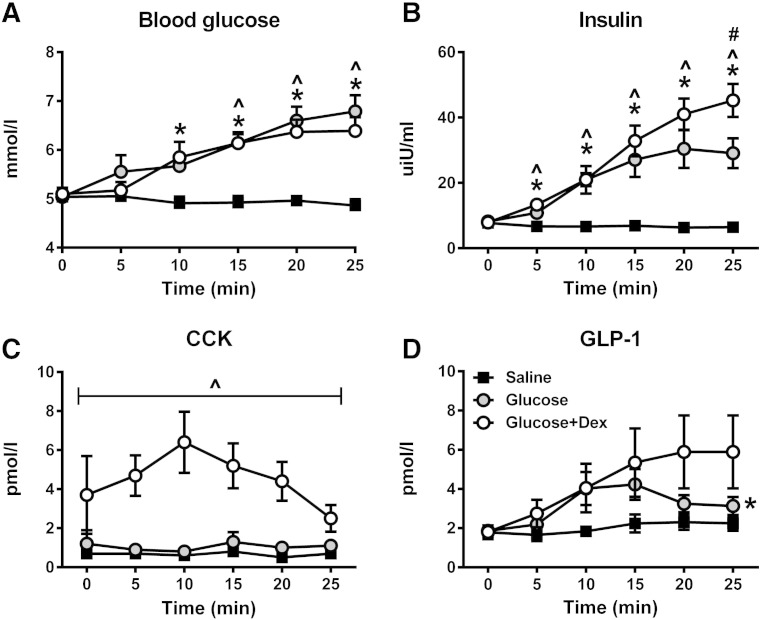
Blood glucose (A) and plasma insulin (B), cholecystokinin (CCK) (C) and glucagon-like peptide-1 (GLP-1) (D) concentrations following bolus intragastric glucose (1000 mOsmol), glucose + dexloxiglumide, or saline (0.9%). *glucose vs. saline, p < 0.01, ^glucose + dex vs. saline, p < 0.001, #glucose vs. glucose + dex, p < 0.05, n = 10.

There was an effect of treatment on blood glucose concentrations (p = 0.002). Both glucose (p = 0.001) and glucose + dex (p = 0.009) increased blood glucose concentrations when compared with saline, with no difference between glucose and glucose + dex (p = 1.000) (Fig. 5A).

#### Insulin

There was an effect of treatment on the AUC for serum insulin concentrations (p < 0.001). Both glucose (p = 0.003) and glucose + dex (p = 0.001) increased serum insulin concentrations when compared with saline. There was a treatment ∗ time interaction (p < 0.001). Insulin concentrations were greater for glucose and glucose + dex between t = 5–25 min, when compared with saline, and greater for glucose + dex when compared with glucose at t = 25 min (Fig. 5B).

#### CCK

There was an effect of treatment on the AUC for plasma CCK concentrations (p = 0.002). CCK was increased following glucose + dex when compared with glucose (p = 0.005) and saline (p = 0.007), with no differences between glucose and saline (p = 0.395) (Fig. 5C).

#### GLP-1

There was an effect of treatment on the AUC for plasma GLP-1 concentrations (p = 0.001) (Fig. 5D). Glucose increased plasma GLP-1 concentrations when compared with saline (p = 0.047), with no difference between glucose and glucose + dex, or glucose + dex and saline.

### Perceptions of hunger, fullness and nausea

**Fig. 6:**
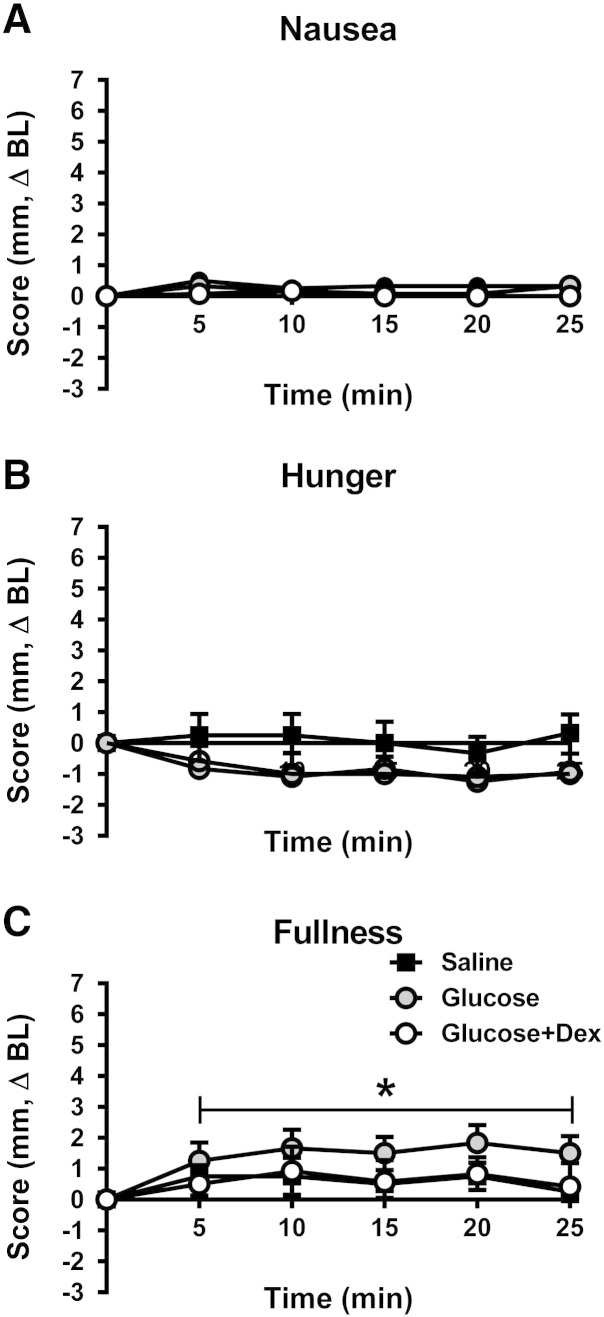
Effects of saline, glucose and glucose + dexloxiglumide on perceptions of nausea (A), hunger (B) and fullness (C). There were no differences in baseline ratings, nor any effect of any treatment, on perceptions of nausea or hunger. There was an effect of treatment for fullness scores (p = 0.05). Glucose increased fullness when compared with saline (p = 0.009), with no differences between saline and glucose + dex. *P < 0.05 glucose vs. saline.

There were no differences in baseline ratings, nor any effect of any treatment, on perceptions of nausea (Fig. 6A) or hunger (Fig. 6B). There was an effect of treatment for fullness scores (p = 0.05). Glucose increased fullness when compared with saline (p = 0.009), with no differences between saline and glucose + dex (Fig. 6C).

Changes in fullness were not correlated to circulating levels of glucose, CCK, GLP-1 or insulin. Ratings of hunger were weakly related to plasma insulin (R = 0.36, P = 0.052), but not CCK, glucose or GLP-1, concentrations.

## Discussion

The development of whole brain fMRI applied to intragastric infusion of nutrients (physiological MRI; physMRI) has provided an entirely new means to understand the gut-to-brain signalling mechanisms regulating gastrointestinal function, appetite and food intake in humans (Jones et al., 2012, Lassman et al., 2010). We have now, for the first time, demonstrated the whole brain responses to glucose when administered directly into the stomach to avoid the confounding effects of sight, taste or smell, and overcoming the technical issues associated with movement during swallowing. In the medulla, pons, and midbrain regions of the brainstem and in the hypothalamus (and other areas outlined in Table 1) glucose, compared to the saline, decreased the BOLD signal at 5 min, after a small initial increase. The brainstem and hypothalamic regions have previously been identified to respond to intragastric lipid, using identical methods (Jones et al., 2012, Lassman et al., 2010). However, in response to glucose, the decrease in BOLD signal was temporally correlated to the increases in blood glucose and plasma insulin concentrations.

Since CCK clearly mediated gastric and gall bladder emptying following intragastric glucose, the role of CCK in the CNS response to intragastric infusion of glucose was investigated using a pretreatment with dexloxiglumide (a CCK_1_ receptor antagonist). Investigation of the interaction between glucose and glucose + dex over time revealed several interesting results. There was a partial blocking of the effect of glucose to decrease BOLD signal in the hypothalamus by dexloxiglumide. Dexloxiglumide furthermore abolished the effect of glucose in the motor cortex, the only region to show a BOLD signal increase in response to glucose. In the cerebellum, left caudate, bilateral cuneus and left lingual gyrus, dexloxiglumide pre-treatment further reduced the glucose BOLD response. Interestingly, there was a significant temporal correlation with insulin and GLP-1 in all these regions apart from the left caudate. For the remaining regions dexloxiglumide changed the temporal characteristics of the glucose response by abolishing the initial increase in BOLD signal as observed for glucose vs. saline.

With regard to the areas showing decreased BOLD signal, our observations confirm previous reports of a decrease in BOLD signal within the hypothalamus following either oral or intravenous glucose in humans (Liu et al., 2000, Matsuda et al., 1999, Smeets et al., 2005a, Smeets et al., 2005b, Smeets et al., 2007, Vidarsdottir et al., 2007), and rats (Chen et al., 2007, Tsurugizawa et al., 2008), and for the first time define the brainstem response to the presence of glucose in the GI tract in humans. In seeking to explore candidate pathways for these effects of glucose to decrease BOLD signal in the CNS regions outlined in Table 1, our dexloxiglumide data indicate that, unlike the response to lipid (Lassman et al., 2010), the response to glucose in these regions is not solely dependent on the activation of CCK_1_ receptors on vagal afferents. Even though we demonstrated that glucose does activate processes known to be CCK-dependent (i.e. dexloxiglumide reversible gallbladder contraction and slowing of gastric emptying (Fried et al., 1991, Little et al., 2010, Moran et al., 1997)), there was no significant effect of dexloxiglumide on BOLD signal reduction in these regions. This suggests that although glucose is likely to have activated a subset of neurons via the CCK_1_ receptor (as demonstrated by the dexloxiglumide sensitive increase in BOLD signal in cortical areas and possibly the small initial rise in BOLD signal observed prior to the decrease in the brainstem), there was also an additional, perhaps more direct, inhibitory action of circulating glucose and insulin, which may have occurred at the level of peripheral vagal afferents, and/or the brainstem and hypothalamus. This is supported by the observation that in rats, BOLD signal responses in the lateral hypothalamus, as well as other brain regions, to post-oral glucose were the same in both intact and vagotomised rats, whereas BOLD signal responses to glutamine were attenuated in vagotomised rats. This suggests that specific responses occur to different types of nutrients, with vagal activity being critical for the sensing of glutamine and lipid (Lassman et al., 2010), and humoral factors, such as insulin, as well as changes in circulating glucose levels, being important for glucose detection (Tsurugizawa et al., 2009). To this effect a direct comparison of our independent studies using intragastric lipid and glucose has been provided as supplementary data.

It is recognised that elevated glucose levels can directly influence the activity of specific glucose-sensing neurons in hypothalamic and brainstem regions (Levin et al., 2004), and that there are subsets of neurons in which elevated glucose levels are either excitatory or inhibitory (Grabauskas et al., 2010, Levin et al., 2004). In addition, elevated blood glucose may have been signalling to the brain via activation (excitation or inhibition) of peripheral glucose-sensing neurons in the nodose ganglia (Grabauskas et al., 2010). Animal studies strongly indicate that vago-vagal pathways play an important role in the regulation of gastrointestinal motility (Zhou et al., 2008) and eating behaviour (Grabauskas et al., 2008) in response to acute changes in blood glucose levels. Additionally, acute increases in glucose levels modulate synaptic connections between vagal afferent nerve terminals and neurons in the nucleus tractus solitarius (Wan and Browning, 2008). However, while one study has demonstrated an effect of intravenous glucose on BOLD signal in the hypothalamus (Smeets et al., 2007), this effect was not replicated by others, despite inducing comparable increases in blood glucose concentrations (Purnell et al., 2011). A number of studies have reported no relationship between blood glucose concentrations and glucose-induced changes in BOLD signal in the hypothalamus (Smeets et al., 2005a, Smeets et al., 2007). Here, we have demonstrated a strong correlation between the timing of changes in blood glucose and plasma insulin levels, and the decrease in BOLD signal, suggesting that changes in circulating glucose and insulin concentrations are likely to be key mediators of the central response to glucose, rather than direct vagal afferent signalling from the gut.

While we did demonstrate that dexloxiglumide accelerated gastric emptying of glucose, this was not of a sufficient magnitude to modulate circulating glucose or insulin levels, and indeed, in the CNS regions in which we observed a significant relationship with glucose and insulin (i.e. those regions in which BOLD signal decreased), there was no difference in effect between glucose and glucose + dex conditions. Hence, gastric emptying rate is unlikely to be a major determinant of the CNS response reported here.

It appears from our data that the onset of the BOLD signal rise in the motor cortex was rapid, occurring within 5 min post-infusion and before any measurable elevation of plasma glucose. Further analysis of the direct comparison between glucose and lipid BOLD responses, using a two-way repeated measures ANOVA with nutrient (vs. saline) as a between subject factor and time as a within subject factor, highlighted the spatial similarity and temporal interaction between glucose and lipid (Jones et al., 2012, Lassman et al., 2010) related changes in BOLD signal (Supplementary material). The data included in this comparison were collected in parallel on the same scanner with the same imaging parameters during the same time period. The motor cortex was not significant in this analysis due to a lack of direct overlap in the motor cortex for glucose and lipid however the time course for lipid from the motor cortex in Fig. 3D from Lassman et al., 2010 and in Fig. 4A in this paper are very similar (Supplementary Fig. 1A). Previous studies have also demonstrated that cortical areas (including the motor cortex) display an increase in BOLD signal during intravenous glucose (Purnell et al., 2011). That this response was blocked by dexloxiglumide suggests that the early changes in BOLD signal were mediated by a peripheral vagal CCK_1_ receptor-dependent pathway. This is supported by a recent study that identified a positive relationship between changes in BOLD signal in the thalamus, amygdala, caudate, insula and orbitofrontal cortex with changes in plasma CCK concentrations following oral glucose ingestion (Li et al., 2012). The current study however went beyond pure observation and utilised the CCK_1_ receptor antagonist dexloxiglumide, which we have previously shown to abolish the effect of lipid. Therefore this correlation cannot be causal: the fact that BOLD changes are in opposing directions in our study would make an identical signalling molecule driving both rather implausible. We validated that CCK activity was fully blockaded in our study by demonstrating the abolition of glucose-induced gallbladder contraction: measuring peripheral blood levels of CCK is clearly a poor assay of biological activity in this system, since much of the effects of CCK occur at a paracrine level within the gut. Where possible, physiology is better explored by using interventions such as receptor blockade.

It therefore appears that the effects of intestinal glucose are mediated by at least two nutrient sensing pathways in the CNS, both of which may modify appetite, eating behaviour and digestive function either directly (e.g. central detection of circulating insulin/glucose concentrations) or indirectly (e.g. activation of vagal afferents via gut peptides).

It should also be noted that intragastric infusion, while having benefits of ensuring consistent rate of nutrient delivery to the intestine and allowing a pure focus on gut-to-brain signals, does not entirely reflect normal meal ingestion due to the exclusion of oral and cephalic phases. For example, our analyses did not reveal relationships between BOLD signal changes with appetite perceptions. These analyses are likely to be underpowered since we were unable to collect blood from two participants while they were in the scanner. Additionally, subjective ratings of hunger and fullness in several subjects showed no change from pre-infusion baseline and, therefore, could not be correlated with BOLD responses. We ended up with data for n = 6 for hunger, and n = 7 for fullness, in these models. In the included subjects, the increase in fullness and reduction in hunger were very small in magnitude and blood and VAS scores were collected at 5 min intervals, therefore there was less temporal resolution in comparison to the initial BOLD analysis which was done using 2 minute time bins. The small magnitude of change in VAS scores was not unexpected, since it is well established that intragastric infusion has less potent effects on appetite perceptions than oral consumption (Cecil et al., 1998). Future work using this paradigm should investigate the effects of these additional components during meal ingestion.

Of interest, it has recently been shown that GLP-1 in response to lipid is inhibited by dexloxiglumide indicating dependence on CCK receptor activation (Beglinger et al., 2010). This dependence does not appear to be the case in response to glucose stimulation and probably implicates a direct action of glucose on GLP-1 producing enteric mucosal cells.

The other brain regions responding to glucose, including the cerebellum, thalamus, precuneus and motor cortex have been consistently demonstrated in our previous studies to respond to nutrient ingestion and or gut hormones (i.e. ghrelin) (Jones et al., 2012, Lassman et al., 2010), suggesting that they play a role in mediating the gastrointestinal and appetite responses to ingested food. In particular, the cerebellum has been shown to play an important role in food intake (Zhu and Wang, 2008).

In conclusion this study provides important new information about whole brain responses to intragastric glucose in man, and demonstrates that multiple mechanisms operate to modulate BOLD signal in the nutrient-induced brain matrix.

## Disclosures

TJL, SM, RBJ, RMC, JT and DGT have no conflict of interest to declare. MD is an employee of Rottapharm, Monza, Italy.

## Contribution of the authors

TJL: overall organisation and performance of the study, protocol design, data analysis, manuscript preparation.

SMc: physMRI protocol design and data analysis, drafting of the manuscript.

RBJ: conducting the study, data analysis and drafting of the manuscript.

MD: supply of study drug, protocol design, drafting of the manuscript.

JMcL and DGT: secured grant, protocol design, data interpretation, and drafting of the manuscript.
