# Supplementary material for: Mapping glucose-mediated gut-to-brain signalling pathways in humans
Source: Neuroimage. 2014 Aug 1;96:1–11. doi: 10.1016/j.neuroimage.2014.03.059 (PMC4075342; doi:10.1016/j.neuroimage.2014.03.059)
Supplement: Supplementary file 1 — Supplementary Tables and Figures. [file mmc1.docx]

**SUPPLEMENTARY DATA**

**Supplementary Table 1: Significant CNS clusters exhibiting main effect of lipid and glucose ingestion over time at p(FWEc<0.05).** TD – Talairach Daemon; k – number of voxels in cluster; pFWEc – cluster size Family Wise Error corrected p-value; qFDRc – cluster size False Discovery Rate q-value; CoM – centre of mass; x,y,z {mm} – Montreal Neurological Institute (MNI) coordinates from AC-PC origin.

| **main effect of time** | **cluster statistics** | | | **CoM** | | |
| --- | --- | --- | --- | --- | --- | --- |
| **TD Lobes & Hypothalamus** | **k** | **pFWEc** | **qFDRc** | **x,y,z {mm}** | | |
| Brainstem - Medulla | 72 | 0.013 | 0.007 | 1 | -44 | -47 |
| Brainstem - Midbrain L | 70 | 0.015 | 0.008 | -4 | -36 | -21 |
| Brainstem - Midbrain R | 55 | 0.046 | 0.021 | 6 | -33 | -19 |
| Brainstem - Pons L | 72 | 0.013 | 0.007 | -6 | -38 | -28 |
| Brainstem - Pons R | 132 | <0.001 | <0.001 | 10 | -38 | -30 |
| Cerebellum_Ant_L | 534 | <0.001 | <0.001 | -13 | -53 | -28 |
| Cerebellum_Ant_R | 277 | <0.001 | <0.001 | 16 | -43 | -26 |
| Cerebellum_Post_L | 458 | <0.001 | <0.001 | -21 | -60 | -43 |
| Cerebellum_Post_R | 59 | 0.034 | 0.017 | 24 | -58 | -44 |
| Hypothalamus L | 67 | 0.019 | 0.010 | -3 | -3 | -8 |
| Hypothalamus R | 125 | <0.001 | <0.001 | 4 | -3 | -11 |
| **AAL Regions** |  |  |  |  |  |  |
| Cingulum_Mid_L | 55 | 0.046 | 0.021 | -11 | -40 | 40 |
| Cingulum_Mid_R | 97 | 0.002 | 0.001 | 11 | -30 | 38 |
| Cuneus_L | 77 | 0.009 | 0.005 | -15 | -63 | 24 |
| Cuneus_R | 47 | 0.082 | 0.036 | 16 | -66 | 31 |
| Lingual_L | 252 | <0.001 | <0.001 | -14 | -68 | -3 |
| Lingual_R | 108 | <0.001 | <0.001 | 13 | -69 | -1 |
| Precuneus_L | 61 | 0.030 | 0.015 | -12 | -67 | 35 |
| Precuneus_R | 212 | <0.001 | <0.001 | 14 | -55 | 24 |
| SupraMarginal_R | 70 | 0.015 | 0.008 | 54 | -44 | 28 |
| Temporal_Mid_R | 66 | 0.021 | 0.011 | 55 | -47 | 3 |

**Supplementary Table 2: Significant CNS clusters exhibiting interaction between lipid and glucose ingestion over time at p(FWEc<0.05).** TD – Talairach Daemon; k – number of voxels in cluster; pFWEc – cluster size Family Wise Error corrected p-value; qFDRc – cluster size False Discovery Rate q-value; CoM – centre of mass; x,y,z {mm} – Montreal Neurological Institute (MNI) coordinates from AC-PC origin.

| **nutrient x time interaction** | **cluster statistics** | | | **CoM** | | |
| --- | --- | --- | --- | --- | --- | --- |
| **TD Lobes & Hypothalamus** | **k** | **pFWEc** | **qFDRc** | **x,y,z {mm}** | | |
| Brainstem - Medulla | 68 | 0.028 | 0.014 | 1 | -44 | -47 |
| Brainstem - Midbrain L | 47 | 0.098 | 0.041 | -4 | -36 | -20 |
| Brainstem - Midbrain R | 60 | 0.045 | 0.021 | 5 | -34 | -20 |
| Brainstem - Pons L | 61 | 0.042 | 0.020 | -5 | -39 | -28 |
| Brainstem - Pons R | 117 | 0.001 | 0.001 | 8 | -39 | -30 |
| Cerebellum_Ant_L | 217 | <0.001 | <0.001 | -6 | -49 | -26 |
| Cerebellum_Ant_R | 275 | <0.001 | <0.001 | 13 | -42 | -23 |
| Cerebellum_Post_L | 150 | <0.001 | <0.001 | -29 | -56 | -44 |
| Hypothalamus L | 53 | 0.068 | 0.030 | -1 | 0 | -11 |
| Hypothalamus R | 93 | 0.006 | 0.004 | 4 | -3 | -11 |
| **AAL Regions** |  |  |  |  |  |  |
| Cuneus_L | 75 | 0.018 | 0.010 | -15 | -63 | 24 |
| Cuneus_R | 88 | 0.008 | 0.005 | 15 | -66 | 29 |
| Lingual_L | 130 | <0.001 | <0.001 | -14 | -66 | -2 |
| ParaHippocampal_L | 76 | 0.017 | 0.009 | -25 | -33 | -11 |
| Precuneus_L | 50 | 0.082 | 0.035 | -12 | -67 | 35 |
| Precuneus_R | 259 | <0.001 | <0.001 | 14 | -58 | 24 |

**

**

**Supplementary Figure 1:** Comparison of the changes in blood-oxygenation-level-dependent (BOLD) signal within the medulla (L+R) and motor cortex (L+R) (A), pons (B), midbrain (C) and hypothalamus (D) in response to intragastric infusion of 1000 mOsmol glucose and lipid (lauric acid (C12) 0.05 mol/l, ([Lassman et al., 2010](#_ENREF_16))). Grey circles: Lipid, Black circles: glucose.


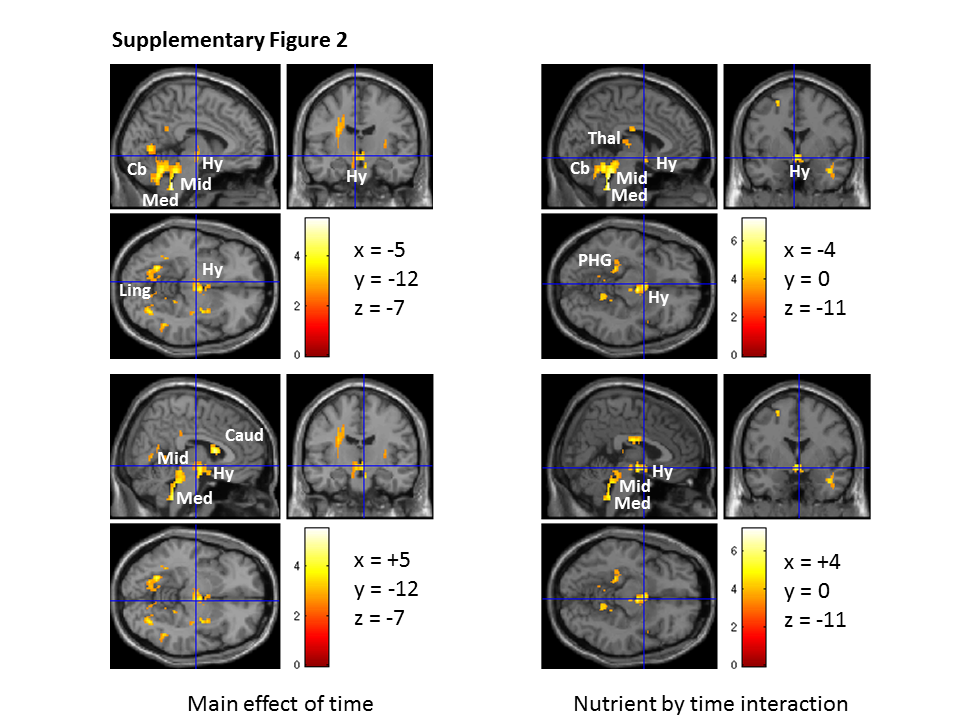


**Supplementary Figure 2**: **Brain overlays showing areas significant clusters exhibiting main effect lipid and glucose ingestion over time and interaction between lipid and glucose ingestion over time at p(FWEc)<0.05.**  Hy: Hypothalamus, Mid: Midbrain, Med: Medulla, Cb: Cerebellum, Thal: Thalamus, Ling: Lingual Gyrus: PHG: Parahippocampal Gyrus, Caud: Caudate. All overlays at p<0.001.
